# Impact of Vaccine-Elicited Anti-Spike IgG4 Antibodies on Fc-Effector Functions Against SARS-CoV-2

**DOI:** 10.3390/v17050666

**Published:** 2025-05-03

**Authors:** Katrina Dionne, Alexandra Tauzin, Étienne Bélanger, Yann Desfossés, Mehdi Benlarbi, Ling Niu, Guillaume Beaudoin-Bussières, Halima Medjahed, Catherine Bourassa, Josée Perreault, Marzena Pazgier, Renée Bazin, Andrés Finzi

**Affiliations:** 1 Département de Microbiologie, Infectiologie et Immunologie, Université de Montréal, Montréal, QC H3T 1J4, Canada; 2 Centre de Recherche du CHUM, Montréal, QC H2X 0A9, Canada; 3 Infectious Disease Division, Department of Medicine, Uniformed Services University of the Health Sciences, Bethesda, MD 20814, USA; 4 Héma-Québec, Affaires Médicales et Innovation, Québec, QC G1V 5C3, Canada

**Keywords:** SARS-CoV-2, humoral responses, IgG4, ADCC, Fc-effector functions, neutralization, elderly donors

## Abstract

mRNA vaccines have demonstrated considerable efficacy and safety against SARS-CoV-2, limiting the pandemic burden worldwide. The emergence of new variants of concern and the decline in neutralizing activity observed several weeks post-vaccination reinforced the call for repeated mRNA vaccination. We and others have shown that vaccine efficacy does not exclusively rely on antibody neutralizing activites; Fc-effector functions play an important role as well. However, it is well known that long-term exposure and repeated antigen stimulation elicit the IgG4 subclass of antibodies, which are inefficient at mediating Fc-effector functions. In this regard, recent studies highlighted concerns about IgG4 induction by mRNA vaccines. Here, we explored the impact of repeated mRNA vaccination on IgG4 induction and its impact on Fc-effector functions. We observed anti-Spike IgG4 elicitation after three doses of mRNA vaccine; the antibody levels further increased with additional doses. Vaccine-elicited IgG4 preferentially bound the ancestral D614G Spike. We also observed that Breakthrough Infection (BTI) after several doses of vaccine strongly increased IgG1 levels but had no impact on IgG4 levels, thereby improving Fc-effector functions. Finally, we observed that elderly donors vaccinated with Moderna mRNA vaccines elicited higher IgG4 levels and presented lower Fc-effector functions than donors vaccinated with the Pfizer mRNA vaccine. Altogether, our results highlight the importance of monitoring the IgG subclasses elicited by vaccination.

## 1. Introduction

The Severe Acute Respiratory Syndrome Coronavirus 2 (SARS-CoV-2) is the etiological agent responsible for the Coronavirus Disease 2019 (COVID-19). As of March 2025, there have been over 777 million confirmed cases of infection, causing over 7 million deaths worldwide [1,2]. SARS-CoV-2 is a single-stranded positive RNA virus and a member of the Betacoronavirus genus [1,3]. It expresses, at its surface, the Spike glycoprotein, a trimer of heterodimers composed of two major subunits: S1, which interacts with the human Angiotensin Converting Enzyme 2 (hACE2) receptor at the surface of the host cell through its Receptor-Binding Domain (RBD), and S2, which harbors the fusion peptide mediating viral and host membrane fusion [3,4,5,6,7]. Thus, Spike is critical for viral entry and the main target of humoral responses.

**Figure 1:**
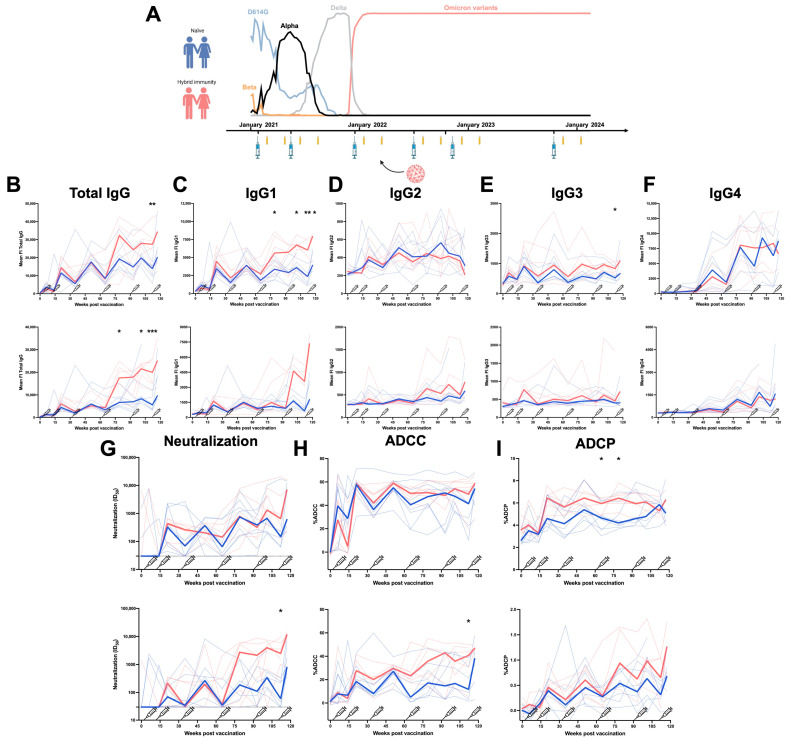
Longitudinal IgG subclasses and humoral responses elicited by mRNA vaccination in a cohort of Health Care Workers. (**A**) Longitudinal SARS-CoV-2 vaccine cohort design, with donors separated into two groups: Naïve (no BTI) and with hybrid immunity (BTI) (data on variants are from INSPQ) [14]. Plasma samples were collected from a cohort of HCW at 4 weeks and 4 months after each dose of mRNA vaccine. CEM.NKr cells stably expressing the D614G or BA.4/5 Spike were stained with plasma samples and a secondary antibody, allowing for the specific detection of (**B**) Total IgG, (**C**) IgG1, (**D**) IgG2, (**E**) IgG3, and (**F**) IgG4, and analyzed by flow cytometry. The values represent the MeanFI (Mean Fluorescent Intensity). (**G**) Neutralization activities were measured by incubating viruses pseudotyped with the indicated Spike (from D614G or BA.4/5 variants) with plasma samples for 1 h at 37 °C before infecting 293T-ACE2 cells. Neutralization half-maximal inhibitory serum dilution (ID_50_) values were determined using a normalized non-linear regression. (**H**) CEM.NKr parental cells mixed with CEM.NKr cells stably expressing the D614G or BA.4/5 Spike at a 1:1 ratio (used as target cells) were incubated with plasma samples and KHYG-1 cells (used as effector cells) to measure ADCC activity in a FACS-based assay. (**I**) CEM.NKr cells stably expressing the D614G or BA.4/5 Spike (used as target cells) were incubated with plasma samples and THP-1 cells (used as effector cells) to measure ADCP activity. Vaccine doses are represented by syringes. Plasma samples collected from HCW with or without BTI are represented by red and blue lines, respectively. Bold lines represent the median for both groups. (* *p* < 0.05; ** *p* < 0.01; *** *p* < 0.001; ns, non-significant).

Shortly after the beginning of the pandemic, two mRNA vaccines (Moderna and Pfizer) based on the ancestral Wuhan Spike were developed and strongly contributed to improve disease outcomes [8,9]. The emergence of various SARS-CoV-2 variants containing numerous mutations in the Spike resulted in increased resistance to the humoral responses elicited by mRNA vaccination [10]. Since the end of 2021, Omicron subvariants are the predominant circulating strains worldwide (Figure 1A) [11,12,13,14]. To improve humoral responses against Omicron (BA.1) and its subvariants, bivalent mRNA-based vaccines composed of mRNA coding for both the ancestral Wuhan and Omicron subvariant Spikes were developed [13,14,15,16,17,18]. This aimed to confer immunity against both variants, but several studies found only a slight improvement in vaccine efficacy [13,16,17].

Immunoglobulin G (IgG) antibodies are important players in vaccine-elicited humoral responses, as they mediate neutralization and Fc-effector functions [19,20]. IgGs are divided into four subclasses (IgG1, IgG2, IgG3, and IgG4). They mainly differ in the sequence of their Fc portion and the length of their hinge region, properties that lead to differences in their biological functions [21]. IgG1 is the most abundant subclass and is a key player in protective immunity, as it mediates strong Fc-effector and neutralizing responses [21]. IgG2 and IgG3 are induced following bacterial or viral infections, and IgG3, with its extended hinge, is known to be involved in potent Fc-effector functions, particularly Antibody-Dependent Cellular Phagocytosis (ADCP) [21,22]. IgG4 is preferentially induced after several exposures to the same antigen as an anti-inflammatory response and has poor affinity for Fc receptors, thereby mediating poor Fc-effector functions [21,23]. Interestingly, several studies reported that IgG4 were strongly induced after several doses of mRNA vaccine (but not other anti-SARS-CoV-2 vaccine platforms) and raised concerns about vaccine efficacy, since millions of individuals received several boosters of the same mRNA vaccine coding for the ancestral Spike or a bivalent version [23,24,25,26].

In this study, we measured the induction of the different IgG subclasses after every dose in a longitudinal cohort of Health Care Workers (HCW) who received six doses of mRNA vaccine. We also evaluated the contribution of every subclass to several parameters of the humoral responses, including Antibody-Dependent Cellular Cytotoxicity (ADCC), ADCP, and neutralization activities against the ancestral D614G strain and the Omicron BA.4/5 subvariant. We also evaluated the impact of Hybrid Immunity (HI), conferred by both vaccination and infection, on the dynamics of IgG subclasses. As vaccination is particularly important for some vulnerable groups, we also measured these responses in a more vulnerable cohort of donors aged over 70 years.

## 2. Materials and Methods

Materials and methods have been previously reported and are summarized below [27,28].

### 2.1. Ethics Statement

This study was conducted in accordance with the Declaration of Helsinki in terms of informed consent and approval by an appropriate board. The protocol was approved by the Ethics Committee of the CHUM (19.381, approved on 28 February 2022) and from plasma donors who consented to participate in the Plasma Donor Biobank at Héma-Québec (PlasCoV; REB-B-6-002-2021-003).

### 2.2. Human Subjects

The longitudinal study was conducted in 14 HCW, 9 females and 5 males (age range: 31–69 years), who received six doses of mRNA vaccine. Blood samples were collected 25 days (median) and 116 days (median) after each dose of mRNA vaccine. Six individuals were infected after the third dose. The characteristics of the groups are summarized in Table 1 and Figure 1A.

**Figure 2:**
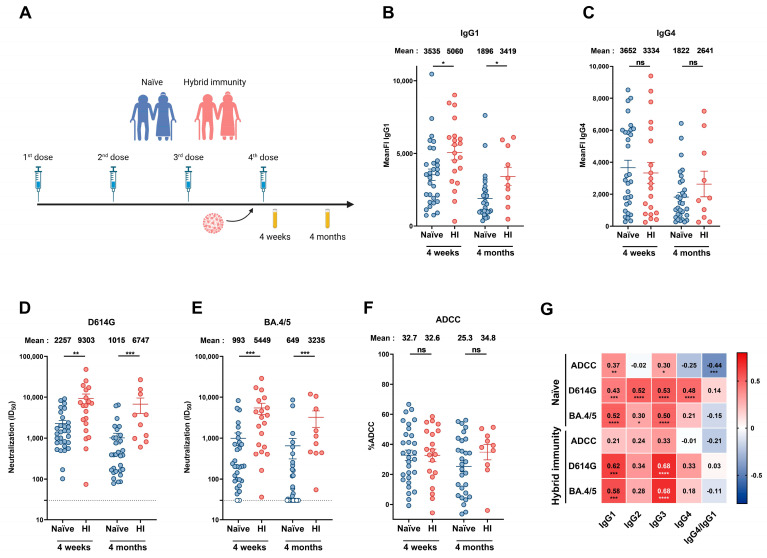
Humoral responses elicited after a fourth dose of mRNA vaccine in people older than 70 years with or without breakthrough infection. (**A**) SARS-CoV-2 vaccine cohort design. Plasma samples were collected from donors older than 70 years with or without BTI at 4 weeks and 4 months after their fourth dose of mRNA vaccine. (**B**,**C**) CEM.NKr cells stably expressing the D614G Spike were stained with plasma samples and a secondary antibody, allowing for the specific detection of (**B**) IgG1 or (**C**) IgG4 subclasses, and analyzed by flow cytometry. The values represent the MeanFI. (**D**,**E**) Neutralization activities were measured by incubating viruses pseudotyped with the indicated Spike (from (**D**) D614G or (**E**) BA.4/5 variants) with plasma samples for 1 h at 37 °C before infecting 293T-ACE2 cells. Neutralization half-maximal inhibitory serum dilution (ID_50_) values were determined using a normalized non-linear regression. (**F**) CEM.NKr parental cells mixed with CEM.NKr cells stably expressing the D614G Spike at a 1:1 ratio (used as target cells) were incubated with plasma samples and KHYG-1 cells (used as effector cells) to measure ADCC activity in a FACS-based assay. (**G**) Spearman correlations between the level of the different IgG subclasses elicited after the fourth dose of mRNA vaccine (4 weeks and 4 months) and the functional activities of the antibodies. Plasma samples collected from donors older than 70 years with or without BTI are represented by red and blue points, respectively. (HI: Hybrid Immunity). Signals below the detection threshold are represented as white symbols, and limits of detection are plotted. Error bars indicate means ± SEM (* *p* < 0.05; ** *p* < 0.01; *** *p* < 0.001; **** *p* < 0.0001; ns, non-significant).

For the elder group, plasma samples were obtained from the Héma-Québec’s PlasCoV biobank [29]. The study was conducted in 79 individuals >70 years old (age range: 70–83 years) who received four doses of mRNA vaccine against SARS-CoV-2 and compared to a group of 19 individuals aged from 30 to 55 years (also from the PlasCoV biobank). For senior donors, blood samples were collected 26 days (median) and 117 days (median) following the second, third, and fourth doses of mRNA vaccine. In this group, 20 individuals were infected by SARS-CoV-2 between the third and fourth doses of mRNA vaccine. The characteristics of the groups are summarized in Table S1 and Figure 2A and Figure S1A. We did not include other specific criteria, such as number of donors (sample size), sex, clinical, or demographic data, for admission to the cohorts.

### 2.3. Plasma Samples and Antibodies

Plasma samples were isolated by Ficoll density gradient from blood samples, heat-inactivated for 1 h at 56 °C, and stored at −80 °C until use. We used plasma samples from SARS-CoV-2 uninfected/unvaccinated donors collected before the pandemic (pre-pandemic) as negative controls in ELISA, ADCC, ADCP, and cytometry assays. The CV3-25 monoclonal antibody (mAb) (a conformationally independent S2-specific mAb) was used as a positive control in ADCC, ADCP, flow cytometry assays, and ELISA against S2 subunit and total Spike [30,31,32]. The CR3022 mAb [33] was used as a positive control against S1 subunit and RBD in ELISA. Alexa Fluor-647-conjugated goat anti-human antibody detecting all IgG isotypes; unconjugated mouse anti-human antibodies detecting, respectively, IgG1, IgG2, IgG3, or IgG4; and an Alexa Fluor-647-conjugated goat anti-mouse antibody detecting all IgGs for flow cytometry experiments or an HRP-conjugated goat anti-human IgG or anti-mouse IgG for ELISA experiments (Invitrogen, Waltham, MA, USA) were used as secondary antibodies. The specificity of the anti-IgG subclass secondary antibodies is illustrated in Figure S2.

### 2.4. Plasmids

The plasmids encoding the SARS-CoV-2 D614G and BA.4/5 Spike variants were previously described, and sequences were verified by Sanger sequencing [11,16,28,34]. The pNL4.3 R-E-Luc plasmid was obtained from the NIH AIDS Reagent Program (cat# 3418). The HPM7 plasmid was kindly provided by Dr. Andrew Ward (The Scripps Research Institute, CA, USA).

### 2.5. Protein Expression and Purification

To produce SARS-CoV-2 Spike RBD WT and S1 proteins, FreeStyle 293 F cells (Invitrogen, Waltham, MA, USA) were transfected with the plasmid coding for SARS-CoV-2 Spike RBD WT or S1 subunit (obtained from SinoBiological, Beijing, China), respectively, using an ExpiFectamine 293 transfection reagent, as directed by the manufacturer (Invitrogen, Waltham, MA, USA). The cells were left at 37 °C for one week before being pelleted, and supernatants were collected. The supernatants were filtered using a 0.22 µm filter (Thermo Fisher Scientific, Waltham, MA, USA). The recombinant RBD and S1 proteins were purified using nickel-affinity columns, as directed by the manufacturer (Invitrogen, Waltham, MA, USA). The purified proteins were then dialyzed against Phosphate-Buffered Saline (PBS). The purity of the recombinant proteins was assessed by staining with Coomassie Blue and loading on SDS-PAGE gels. The RBD proteins were stored in aliquots at −80 °C until further use. The S2 protein was purchased from SinoBiological (SinoBiological, Beijing, China), and the Spike protein was expressed as described previously [35]. Briefly, the SARS-CoV-2 Spike (referred to as HPM7) was designed using the Wuhan strain sequence with six proline mutations (hexaproline or HP) at positions F817P, A892P, A899P, A942P, K986P, and V987P along with an engineered interprotomer disulfide (mut7 or M7) between residues 705 and 883 of the S2 subunit. HPM7 was transiently expressed in 293F GNTI- cells using FectoPRO (Polyplus 116-010). In brief, 50 µg of the HPM7 plasmid and 75 µL of FectoPRO transfection reagent were mixed and incubated at room temperature for 10 min. The transfection mixture was then added to 90 mL of 293F GNTI- cells at a density of approximately 1 million cells/mL. After 4 days of transfection, supernatants were harvested and filtered using a 0.22 μm membrane. HPM7 was initially purified using Strep-Tactin XT resin (IBA Lifesciences, Goettingen, Germany) according to the manufacturer’s instructions, followed by further purification via size-exclusion chromatography (SEC) using Superdex 200 (GE Healthcare, Chicago, IL, USA).

### 2.6. Cell Lines

The 293T and 293T-ACE2 cell lines [36] were maintained in Dulbecco’s Modified Eagle’s Medium (DMEM) (Wisent, Saint-Jean-Baptiste, QC, CA) supplemented with 5% Fetal Bovine Serum (FBS) (VWR, Mississauga, ON, CA) and 100 µg/mL of penicillin–streptomycin (Wisent, Saint-Jean-Baptiste, QC, CA). CEM.NKr CCR5+ cells (NIH AIDS reagent program) and CEM.NKr CCR5 + cells stably expressing the SARS-CoV-2 Spike glycoproteins (D614G or BA.4/5) and the GFP [19,27,37] were maintained in Roswell Park Memorial Institute (RPMI) 1640 medium (GIBCO, Waltham, MA, USA) containing 10% FBS and 100 µg/mL of penicillin–streptomycin. The 293T, 293T-ACE2, and CEM.NKr CCR5 + cells stably expressing the SARS-CoV-2 Spike glycoproteins were maintained at 37 °C under 5% CO_2_. FreeStyle 293F cells were grown in FreeStyle 293F medium (Invitrogen, Waltham, MA, USA) to a density of 10^6^ cells/mL at 37 °C with 8% CO_2_ under regular agitation (135 rpm). KHYG-1 cells [38,39] were maintained in RPMI 1640 medium (GIBCO, Waltham, MA, USA) containing 10% FBS, 2 µg/mL of primocin, 2 µg/mL of cyclosporine A, and 5 U/mL of IL-2. THP-1 cells were maintained in RPMI 1640 medium (GIBCO, Waltham, MA, USA) containing 10% FBS and 100 µg/mL of penicillin–streptomycin.

### 2.7. Anti-Nucleocapsid (N) Assay

A previously described, anti-N antibody levels were measured by ELISA [13,27]. Briefly, 96-well plates were coated with recombinant N (50 µL/well at a concentration of 0.25 µg/mL; Centre National en Électrochimie et en Technologies Environnementales Inc., Shawinigan, QC, Canada) overnight at 4 °C, followed by a blocking step with PBS 2% BSA and 0.1% Tween. Plasma (diluted 1:100 in PBS with 0.1% Tween) was incubated for 1 h at Room Temperature (RT). After washing the plates, anti-human polyvalent IgA + IgG + IgM (H + L)-HRP conjugates were used as the secondary antibody, and the plates were incubated for 1 h at RT. The plates were then washed before adding 100 µL of 3,3′,5,5′-Tetramethylbenzidine (TMB, ESBE Scientific, Markam, ON, CA) and incubated for 20 min at RT. Finally, the colorimetric reaction was stopped by adding 100 µL of H_2_SO_4_ 1N (Fisher Scientific, Waltham, MA, USA) to the plates. The plates were then read within 30 min at 450 nm using a Synergy H1 microplate reader (BioTek, Winooski, VT, USA).

### 2.8. Anti-S ELISA

The SARS-CoV-2 ancestral total S, the S1 and S2 subunits, as well as RBD proteins were prepared at a concentration of 2.5 µg/mL in PBS and plated in 96-well plates (MaxiSorp Nunc, Thermo Fisher Scientific, Waltham, MA, USA) (50 µL/well). Following overnight incubation at 4 °C, a blocking buffer (Tris-Buffered Saline (TBS) containing 0.1% Tween20 and 2% BSA) was added to the coated wells after removing the supernatants and incubated for 1 h at RT. The plates were then washed four times using a washing buffer (TBS containing 0.1% Tween20). After washing, the CR3022 (for S1 and RBD ELISA) and the CV3-25 (for S2 and total Spike ELISA) mAbs at a concentration of 50 ng/mL or plasmas diluted at 1/250 in a solution of blocking buffer (0.1% BSA) were added to coated wells and incubated for 90 min at RT. After washing as previously described, anti-human IgG Fc conjugated to HRP (1/3000) or mouse anti-human IgG1, IgG2, IgG3 (1/1000), or IgG4 (1/250) (Invitrogen, Waltham, MA, USA) unconjugated secondary antibodies were diluted in a solution of blocking buffer (0.4% BSA), added to the correspondent wells, and incubated for 90 min at RT. After washing, anti-mouse IgG-HRP (1/2500) was added to the respective plates (with unconjugated anti-human IgG) in a blocking buffer (0.4% BSA) and incubated for 1 h at RT. After washing the plates four times, HRP enzyme activity was determined by adding a 1:1 mix of Western Lightning oxidizing and luminol reagents (Perkin Elmer Life Sciences, Waltham, MA, USA), and an LB942 TriStar luminometer (Berthold Technologies, Bad Wildbad, Germany) was used to measure light emission. The normalization was calculated with the signal obtained with either CR3022 or CV3-25 mAb. Nine pre-pandemic plasma samples were used to establish the seropositivity threshold, using the following formula: mean of pre-pandemic SARS-CoV-2-negative plasma + (3 standard deviations of the mean of pre-pandemic SARS-CoV-2-negative plasma).

### 2.9. Cell-Surface Staining and Flow Cytometry Analysis

CEM.NKr CCR5+ cells stably expressing the SARS-CoV-2 Spike glycoproteins (D614G or BA.4/5) and GFP were used as target cells to assess the binding capacity of the plasma from vaccinated and/or infected individuals. The cells were stained for 45 min at RT with the CV3-25 mAb (1 µg/mL) as the control or with plasma diluted in PBS (1/1000 dilution for total IgG and 1/500 dilution for IgG subclasses). The cells were then washed with PBS and stained with a secondary Ab (Alexa Fluor-647-conjugated goat anti-human IgGs diluted in PBS (1/1000) or unconjugated mouse anti-human IgG1 (1/500), IgG2 (1/2000), IgG3 (1/1000), IgG4 (1/2000) diluted in PBS) for 20 min at RT. For the IgG subclasses staining, the cells were then washed with PBS and stained with a tertiary Ab (Alexa Fluor-647-conjugated goat anti-mouse IgG diluted in PBS (1/1000) for 20 min at RT. The cells were washed twice in PBS and fixed in a solution of paraformaldehyde (PFA) 2% before being passed on a cytometer. All samples were acquired on an LSRII cytometer (BD Biosciences, Mississauga, ON, Canada). The percentage of Spike-expressing cells (GFP+ cells) was determined by gating the living cell population based on viability dye staining (Aqua Vivid, Invitrogen, Waltham, MA, USA). We performed data analysis using FlowJo v10.8.0 (Tree Star, Ashland, OR, USA) by gating on the alive GFP+ population.

### 2.10. Antibody-Dependent Cellular Cytotoxicity Assay

This assay was previously described [19,37]. Briefly, parental CEM.NKr CCR5+ cells were mixed at a 1:1 ratio with the different transduced cell lines (CEM.NKr.Spike D614G or CEM.NKr.Spike BA.4/5). Target cells were marked with a cellular dye (cell proliferation dye eFluor670; Thermo Fisher Scientific, Waltham, MA, USA) and stained for viability (Aqua Vivid, Invitrogen, Waltham, MA, USA). KHYG-1 cells were used as effector cells and stained with a different cellular marker (cell proliferation dye eFluor450; Thermo Fisher Scientific, Waltham, MA, USA) [38]. The stained effector and target cells were mixed in 96-well V-bottom plates at a ratio of 5:1. Plasma samples from pre-pandemic and vaccinated individuals (1/1000) or 1 µg/mL of monoclonal antibody CV3-25 WT or bearing the GASDALIE or LALA mutations (as internal controls of experiments) were added to the appropriate wells. After resuspension, the plates were centrifuged for 1 min at 300× *g* and incubated at 37 °C, 5% CO_2_ for 5 h. To stop the ADCC assay, 100 µL of a solution of PFA 4% (final concentration: 2% PFA) was added to the wells. Since transduced CEM.NKr.Spike D614G and BA.4/5 cells express GFP, ADCC activity was calculated using the following formula: [(% of GFP + cells in Targets plus Effectors) − (% of GFP + cells in Targets plus Effectors plus plasma or antibody)]/(% of GFP + cells in Targets) × 100 by gating on live target GFP+ cells. All samples were acquired on an LSRII cytometer (BD Biosciences, Mississauga, ON, Canada), and data analysis was performed using FlowJo v10.8.0 (Tree Star, Ashland, OR, USA).

### 2.11. Antibody-Dependent Cellular Phagocytosis Assay

For evaluation of anti-SARS-CoV-2 ADCP activity, CEM.NKr CCR5+ cells stably expressing the SARS-CoV-2 Spike glycoproteins (D614G or BA.4/5) were used as target cells, as they were marked with a cellular dye (cell proliferation dye eFluor450; Thermo Fisher Scientific, Waltham, MA, USA) and stained for viability (AquaVivid). THP-1 cells were stained with a different cellular marker (cell proliferation dye eFluor670; Thermo Fisher Scientific, Waltham, MA, USA) and used as effector cells. The stained target cells were mixed with plasma samples from pre-pandemic and vaccinated and/or infected individuals (1/500) or 5 µg/mL mAb CV3-13 WT, GASDALIE, or LALA or CV3-25 WT, GASDALIE, or LALA (as internal controls of experiments) in RPMI in 96-well U-bottom plates and incubated for 1 h at 37 °C. The cells were then washed twice with RPMI medium, and the stained effector cells were added to the mix at a final ratio of 1 effector:5 target cells. The plates were incubated at 37 °C, 5% CO_2_ for 5 h before washing twice with RPMI and adding 100 µL of a solution of PFA 2%. ADCP activity was measured by gating on GFP+ transduced live target cells. All samples were acquired on an LSRII cytometer (BD Biosciences, Mississauga, ON, Canada), and data analysis was performed using FlowJo v10.8.0 (Tree Star, Ashland, OR, USA).

### 2.12. Pseudovirus Neutralization Assay

293T cells were transfected with the lentiviral vector pNL4.3 R-E− Luc and a plasmid encoding the D614G or the BA.4/5 Spike glycoprotein at a ratio of 10:1 to produce SARS-CoV-2 pseudoviruses bearing the corresponding Spike. Cell supernatants were harvested two days after the transfection and stored at −80 °C until use. Regarding the neutralization assay, 293T-ACE2 cells were used as target cells and were seeded at a density of 1 × 10^4^ cells/well in 96-well luminometer-compatible tissue culture plates (PerkinElmer, Waltham, MA, USA) 24 h before infection with pseudoviruses. The next day, pseudoviral particles were incubated with several plasma dilutions (1/50; 1/250; 1/1250; 1/6250; 1/31250) for 1 h at 37 °C and were then added to the target cells. The plates were incubated for 48 h at 37 °C. After 48 h, the supernatant in each well was removed, and 30 µL of lysis buffer 1X (Promega, Madison, WI, USA) was added to the wells to lyse the cells, which was followed by one freeze–thaw cycle. The luciferase activity of each well was measured using an LB942 TriStar luminometer (Berthold Technologies, Bad Wildbad, Germany) by adding 100 µL of luciferin buffer (15 mM MgSO_4_, 15 mM KH_2_PO_4_ [pH 7.8], 1 mM ATP, and 1 mM dithiothreitol) and 50 µL of 1 mM d-luciferin potassium salt (Prolume, Randolph, VT, USA). The neutralization half-maximal inhibitory dilution (ID_50_) represents the plasma dilution to inhibit 50% of the infection of 293T-ACE2 cells by pseudoviruses bearing the D614G or BA.4/5 Spike. Neutralization half-maximal inhibitory concentration (ID_50_) values were determined using a normalized non-linear regression using GraphPad Prism version 9.5.1 (GraphPad, San Diego, CA, USA).

### 2.13. Statistical Analysis

We used GraphPad Prism version 9.5.1 (GraphPad, San Diego, CA, USA) to analyze our data. In all the figures, every point and every line represent a biologically independent sample. We tested every dataset for statistical normality and subsequently applied the appropriate (parametric or nonparametric) statistical test. The *p* values < 0.05 were considered significant; significance values are indicated as * *p* < 0.05, ** *p* < 0.01, *** *p* < 0.001, **** *p* < 0.0001, and ns., non-significant.

## 3. Results

### 3.1. Longitudinal Humoral Responses Elicited by mRNA Vaccination in a Cohort of Health Care Workers

To evaluate the humoral responses elicited by mRNA vaccination over time, we collected plasma samples from 14 HCW (nine females and five males) who received six doses of anti-SARS-CoV-2 mRNA vaccine. The plasmas were sampled at 4 weeks and 4 months after every dose, and six of the fourteen individuals were infected between the third and fourth doses of vaccine by an Omicron variant circulating at this time in Montreal, Canada. Breakthrough Infection (BTI) was determined by measuring anti-N antibodies in plasma. The characteristics of the cohort are summarized in Table 1 and Figure 1A.

We first measured the levels of the different IgG subclasses and total IgG recognizing the D614G or the BA.4/5 Spikes in the plasma of the HCW (Figure 1B–F). When measuring the level of total IgG, we observed that the third dose of mRNA vaccine induced strong levels of IgGs recognizing the D614G Spike (and to a lesser extent BA.4/5 Spike). Additional doses did not improve this level of recognition in naïve donors (Figure 1B). However, BTI between the third and fourth doses significantly improved the level of total IgG recognizing both Spikes (Figure 1B). We observed the same tendency for the IgG1 subclass, with levels stabilizing after the third dose of mRNA vaccine and a significantly higher level of IgG1 recognizing both Spikes in individuals infected after their third dose of vaccine (Figure 1C). In contrast, low levels of IgG2 and IgG3 were induced by mRNA vaccination, and BTI only slightly improved the level of IgG3 but not IgG2. Contrary to the other subclasses, the IgG2 levels declined after the sixth dose (Figure 1D,E).

A strong induction of IgG4 in both naïve individuals and donors with BTI was observed after the third dose of mRNA vaccine. No differences were observed between donors with or without BTI (Figure 1F). Of note, IgG4 recognized the D614G Spike better than the BA.4/5 Spike.

We also noted that individuals with hybrid immunity (i.e., who were vaccinated and then infected) had higher levels of IgG1 subclasses against different domains of the Spike (S1, S2, and RBD) compared to naïve donors (Figure S3). Anti-Spike IgG3 appeared after earlier doses of vaccination, while IgG2 as well as IgG4 appeared after the third dose of mRNA vaccine. Vaccine-elicited IgG2 and IgG4 recognized the full Spike as well as S1 and S2 subunits and the RBD (Figure S3), suggesting that all domains of the Spike are sufficiently seen by the immune system during mRNA vaccination in order to elicit IgG4.

We also measured the functional activity of all collected samples (Figure 1G–I). The first two doses of mRNA vaccine induced a high level of antibodies neutralizing D614G pseudoviruses, and additional doses reached the same level of neutralizing antibodies. BTI improved, albeit not significantly, the neutralization activity against the D614G variant. In contrast, poor neutralizing activity against the BA.4/5 variant was elicited by vaccination in naïve donors, with BTI significantly improving it (Figure 1G). ADCC responses against the D614G Spike were observed after the second dose of mRNA vaccine and remained stable over time despite additional doses or BTI. In contrast, low ADCC activity was measured against the BA.4/5 Spike after vaccination but significantly improved in infected individuals after the fifth dose of mRNA vaccine (Figure 1H). The same tendency was observed for the ADCP response (Figure 1I). These results suggest that vaccination induced strong neutralization, ADCC, and ADCP against D614G but induced a weaker response against BA.4/5. In contrast, an infection with an Omicron subvariant enhanced these humoral responses against the Omicron Spike BA.4/5.

Finally, we also measured the associations between the levels of the different IgG subclasses and the functional activity. IgG1 levels are overall positively associated with the ADCC response against D614G and BA.4/5 for both groups of HCW (Figure S4). This strong association is lost after doses five and six, with the concomitant increase in anti-Spike IgG4 levels (Figure S4). Accordingly, the IgG4/IgG1 ratio was negatively associated with the ADCC response against the D614G and BA.4/5 Spikes after a repeated mRNA vaccination in both groups of HCW (Figure S4). IgG4 levels were positively associated with the neutralizing response against the D614G and BA.4/5 Spikes (Figure S4). These results suggest that IgG1 is crucial in viral clearance, as it positively contributes to humoral responses, and although IgG4 has neutralizing properties, it is negatively associated with Fc-effector functions.

### 3.2. Humoral Responses Elicited in Donors Aged over 70 Years

Aged individuals are known to respond less efficiently to vaccines [40,41]. However, recent studies have shown that the difference in antibody responses to COVID-19 vaccination between age groups narrows as the number of doses increases [42,43]. Despite these findings, the production of specific IgG subclasses in vaccinated elderly individuals remains unexplored. To investigate whether repeated mRNA vaccination also elicited anti-Spike IgG4 antibodies in senior individuals, plasma samples were collected from a cohort of donors aged 70 years and older. First, we compared humoral responses in senior donors to a group of individuals aged from 30 to 55 years (basic demographic characteristics of the two groups are detailed in Table S1 and Figure S1A). As expected, after the second dose of mRNA vaccine, donors aged between 30 and 55 years elicited higher levels of the different IgG subclasses, especially IgG1 and IgG3, associated with higher ADCC and neutralizing responses (Figure S1B–H). However, these differences were no longer observed after the third dose of mRNA vaccine. Surprisingly, we observed that donors aged over 70 years elicited more IgG4 than younger donors after the third dose of mRNA vaccine (Figure S1E). IgG4 levels were negatively associated with ADCC responses (Figure S1H,I).

We also measured the impact of BTI on humoral responses elicited in donors aged over 70 years (Figure 2). For this, anti-N ELISA was performed regularly from the beginning of the vaccine campaign in our cohort of senior donors to identify donors who had been infected. We analyzed humoral responses at 4 weeks and 4 months after the fourth dose of mRNA vaccine in individuals that have never been infected or in donors infected just before receiving their fourth dose of vaccine (Figure 2A and Table S2).

When looking at the different IgG subclasses, we observed higher levels of IgG1 and IgG3 in donors who had a BTI (Figure 2B and Figure S5B). No significant differences were observed in IgG2 and IgG4 levels between naïve donors and donors with hybrid immunity (Figure 2C and Figure S5A). While donors who had breakthrough infections exhibited stronger neutralizing responses than naïve donors (Figure 2D,E), we did not observe significant differences in ADCC after the fourth dose (Figure 2F).

Finally, we calculated the association between every IgG subclass and the humoral responses elicited in naïve and BTI donors after the fourth dose of vaccine. IgG1 and IgG3 levels were positively associated with the neutralization response against the D614G and BA.4/5 Spikes in both groups (Figure 2G). In naïve donors, ADCC correlated with IgG1 and IgG3 (Figure 2G). These results suggest that following mRNA vaccination in elderly individuals, not only is IgG1 crucial for viral clearance, but also IgG3. In contrast, IgG4 might act as an inhibitor of the ADCC response.

### 3.3. Impact of Vaccine Platform on Humoral Responses

**Figure 3:**
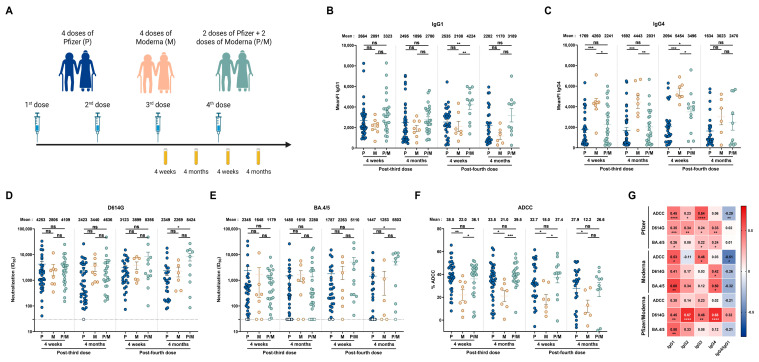
The vaccine platform impacts humoral responses elicited in people older than 70 years. (**A**) SARS-CoV-2 vaccine cohort design. Plasma samples were collected from donors older than 70 years who were vaccinated with 4 doses of Pfizer (P), 4 doses of Moderna (M), or 2 doses of Pfizer and 2 doses of Moderna (P/M). The samples were collected at 4 weeks and 4 months after their third and fourth doses of mRNA vaccine. (**B**,**C**) CEM.NKr cells stably expressing the D614G Spike were stained with plasma samples and a secondary antibody, allowing for the specific detection of (**B**) IgG1 or (**C**) IgG4 subclasses, and analyzed by flow cytometry. The values represent the MeanFI. (**D**,**E**) Neutralization activities were measured by incubating viruses pseudotyped with the indicated Spike (from (**D**) D614G or (**E**) BA.4/5 variants) with plasma samples for 1h at 37 °C before infecting 293T-ACE2 cells. Neutralization half-maximal inhibitory serum dilution (ID_50_) values were determined using a normalized non-linear regression. (**F**) CEM.NKr parental cells mixed with CEM.NKr cells stably expressing the D614G Spike at a 1:1 ratio (used as target cells) were incubated with plasma samples and KHYG-1 cells (used as effector cells) to measure ADCC activity in a FACS-based assay. (**G**) Spearman correlations between the level of the different IgG subclasses elicited after the third and fourth doses of mRNA vaccine (4 weeks and 4 months) and the functional activities of the antibodies. Plasma samples collected from donors older than 70 years who were vaccinated with 4 doses of Pfizer, 4 doses of Moderna, or 2 doses of Pfizer and 2 doses of Moderna are represented by blue, beige, and green points, respectively. Signals below the detection threshold are represented as white symbols, and limits of detection are plotted. Error bars indicate means ± SEM (* *p* < 0.05; ** *p* < 0.01; *** *p* < 0.001; **** *p* < 0.0001; ns, non-significant).

Finally, we examined if the type of mRNA vaccine platform used (i.e., Pfizer or Moderna) had an impact on humoral responses elicited in senior individuals. Among the donors who never experienced BTI, some received four doses of Pfizer, others received four doses of Moderna, and others received two doses of Pfizer and two doses of Moderna (Figure 3A and Table S3).

We observed no significant differences in the levels of vaccine-elicited IgG1 and IgG3 between individuals vaccinated with the Pfizer or Moderna platforms (Figure 3B and Figure S5D). We noted, however, that individual donors vaccinated with the Moderna mRNA vaccine elicited higher levels of IgG4 and, to a lesser extent, IgG2 than the other groups (Figure 3C and Figure S5C). Pfizer and Moderna vaccines induced similar levels of neutralization against D614G and BA.4/5 pseudoviruses (Figure 3D,E), but seniors vaccinated with the Moderna mRNA vaccine presented lower ADCC responses (Figure 3F).

Finally, we measured the association of each subclass and humoral response elicited after the different vaccine regimens in elderly individuals. IgG1 levels were positively associated with all humoral responses measured in all three groups of vaccinated individuals (Figure 3G). Interestingly, IgG3 levels were positively associated with both neutralizing and ADCC responses in Pfizer and Pfizer/Moderna vaccinated individuals, but lower levels appear to be elicited after four doses of the Moderna vaccine, which could be associated with the lower levels of ADCC observed (Figure 3G). These results suggest that Moderna-vaccinated individuals elicit less IgG3 and more IgG4, decreasing ADCC response against the ancestral strain of SARS-CoV-2.

## 4. Discussion

Rapidly after the beginning of the SARS-CoV-2 pandemic, two mRNA vaccine platforms were deployed worldwide. They were the first approved SARS-CoV-2 mRNA vaccines, and at that time, limited information regarding their efficacy was available. Several years after their first administration, the mRNA vaccine platforms were shown to be safe and highly effective to decrease severe disease and mortality [8,9].

With the emergence of new variants of concern, presenting several levels of resistance to humoral responses and with the rapid waning of antibody responses after vaccine administration, health authorities in many countries were encouraged to administer additional doses to maintain an effective level of protection, particularly in vulnerable populations [11,13,15,27,28]. While these additional doses led to a rapid rebound of anti-Spike antibodies, we and others observed a strong induction of IgG4 after three or more doses of mRNA vaccine [44]. IgG4 is a subclass of the IgG that maintains robust neutralizing capacity but has impaired Fc-effector functions due to its reduced affinity to Fc receptors present at the surface of immune cells, such as FcγRIIIa, an important receptor in ADCC responses induced by monocytes and natural killer cells [23,25,26,27,28].

Several studies previously demonstrated that, among the humoral responses elicited by SARS-CoV-2 vaccination and/or infection, Fc-effector functions play a key role in protection. For example, with no or weak neutralizing responses, ADCC contributes to controlling viral replication in mice transgenic for the human receptor ACE2 [31,34,45]. In the present study, we observed a negative association between the ratio of IgG4/IgG1 and ADCC activities, suggesting that the proportion of the different antibody subclasses play an important role in plasma anti-viral activities. Interestingly, the levels of IgG4 recognizing the Omicron BA.4/5 variant Spike were low compared to D614G. Anti-Spike IgG4 antibodies were similarly elicited after repeated vaccination of senior individuals, with a concomitant decrease in ADCC. Of note, this increase was higher in individuals that received the Moderna mRNA vaccine; this could be due to the higher concentration of SARS-CoV-2 Spike mRNA present in the Moderna vaccine compared to the Pfizer platform [24].

## 5. Conclusions

Our study provides a longitudinal overview of the induction of subclasses and humoral responses following repeated anti-SARS-CoV-2 mRNA vaccination in HCW and a vulnerable population. Altogether, our results highlight the need to monitor anti-viral IgG subclasses of antibodies to inform future vaccine design.

## Figures and Tables

**Table 1:** Characteristics of the health care worker cohort.

		Health Care Workers	
		No Recent BTI	Recent BTI
Number (n) ^a^		8	6
Age ^b^		53.5 (31–69)	58 (51–62)
Sex ^a^	Female (n)	7	2
	Male (n)	1	4
First dose (n) ^a^	Pfizer monovalent	5	6
	Moderna monovalent	3	0
Days between the first and second dose ^b^		97.5	111.5
Second dose (n) ^a^	Pfizer monovalent	5	6
	Moderna monovalent	3	0
Days between the second and third dose ^b^		203	218
Third dose (n) ^a^	Pfizer monovalent	6	5
	Moderna monovalent	2	0
	NA	0	1
Days between the third and fourth dose ^b^		195.5	186.5
Fourth dose (n) ^a^	Pfizer monovalent	8	5
	Moderna monovalent	0	0
	Moderna bivalent BA.1	0	1
Days between the fourth and fifth dose ^b^		111	120
Fifth dose (n) ^a^	Pfizer monovalent	0	1
	Moderna bivalent BA.1	2	1
	Pfizer bivalent BA.4/5	4	3
	NA	1	1
Days between the fifth and sixth dose ^b^		350	378
Sixth dose (n) ^a^	Pfizer monovalent	1	0
	Pfizer bivalent BA.4/5	2	1
	Moderna bivalent BA.1	1	0
	Pfizer monovalent XBB.1.5	1	3
	Moderna monovalent XBB.1.5	2	0

^a^: Values displayed are numbers. ^b^: Values displayed are medians, with interquartile ranges in parentheses. NA: Not available.

## Data Availability

Further information, data reported in this paper, and requests for resources and reagents should be directed to and will be fulfilled by the lead contact, Andrés Finzi (andres.finzi@umontreal.ca), upon request.

## Supplementary Materials

The following supporting information can be downloaded at: https://www.mdpi.com/article/10.3390/v17050666/s1, Table S1. Characteristics of the SARS-CoV-2 vaccinated cohort, related to Figure S1; Table S2. Characteristics of the SARS-CoV-2 naïve donors or experiencing BTI after the fourth dose of mRNA vaccine, related to Figure 2 and S5; Table S3. mRNA vaccine platform used in the senior cohort, related to Figure 3 and S5; Figure S1. Humoral responses elicited after the second and third doses of mRNA vaccine in people older than 70 years compared to younger donors; Figure S2. Binding specificity of secondary anti-IgG1, anti-IgG2, anti-IgG3, and anti-IgG4 antibodies tested by flow cytometry and ELISA; Figure S3. Levels of the different IgG subclasses elicited against different domains of the Spike in a cohort of health care workers; Figure S4. Spearman correlations between the levels of the different IgG subclasses elicited after every dose of mRNA vaccine and the functional activities of the antibodies, related to Figure 1; Figure S5. Levels of IgG2 and IgG3 elicited after mRNA vaccine, related to Figure 2 and Figure 3.
