# Supplementary material for: Impact of Vaccine-Elicited Anti-Spike IgG4 Antibodies on Fc-Effector Functions Against SARS-CoV-2
Source: Viruses. 2025 May 3;17(5):666. doi: 10.3390/v17050666 (PMC12115895; doi:10.3390/v17050666)
Supplement: Supplementary file 1 [file viruses-17-00666-s001.zip › viruses-3598219-supplementary.pdf]

Table S1. Characteristics of the SARS-CoV-2 vaccinated cohort, related to Figure S1.

		30-55 years	>70 years
Number (n) [a]		19	79
Age [b]		42 (31-55)	72 (70-83)
Sex [a]	Female (n)	9	31
	Male (n)	10	48
Days between the first and second dose [b]		66 (62-79)	77 (71-88)
Days between the second and third dose [b]		196 (185-210)	191 (185-202)
Days between the third and fourth dose [b]		280 (172-309)	127 (111-196)

[a]Values displayed are numbers. [b]Values displayed are medians, with interquartile ranges in parentheses.

Table S2. Characteristics of the SARS-CoV-2 naïve donors or experiencing BTI after the fourth dose of mRNA vaccine, related to Figure 2 and S5.

		Naïve	Hybrid immunity
Number (n) [a]		35	20
Age [b]		73 (71-75)	71 (70-73)
Sex [a]	Female (n)	12	8
	Male (n)	23	12
Days between the first and second dose [b]		74 (70-84)	81 (73-89)
Days between the second and third dose [b]		189 (184-196)	193 (187-205)
Days between the third and fourth dose [b]		119 (107-134)	196 (126-242)

[a]Values displayed are numbers. [b]Values displayed are medians, with interquartile ranges in parentheses.

Table S3. mRNA vaccine platform used in the senior cohort, related to Figure 3 and S5.

		>70 years old (naïve)		
		4 doses of Pfizer (monovalent)	4 doses of Moderna (monovalent)	2 doses of Pfizer + 2 doses of Moderna (monovalent)
Number (n) [a]		35	8	25
Age [b]		72 (71-75)	70 (70-75)	73 (71-77)
Sex [a]	Female (n)	12	0	15
	Male (n)	23	8	10
Days between the first and second dose [b]		75 (70-86)	79 (72-92)	79 (72-89)
Days between the second and third dose [b]		189 (184-196)	190 (185-217)	195 (189-205)
Days between the third and fourth dose [b]		125 (110-138)	114 (106-220)	129 (113-209)

[a]Values displayed are numbers. [b]Values displayed are medians, with interquartile ranges in parentheses.

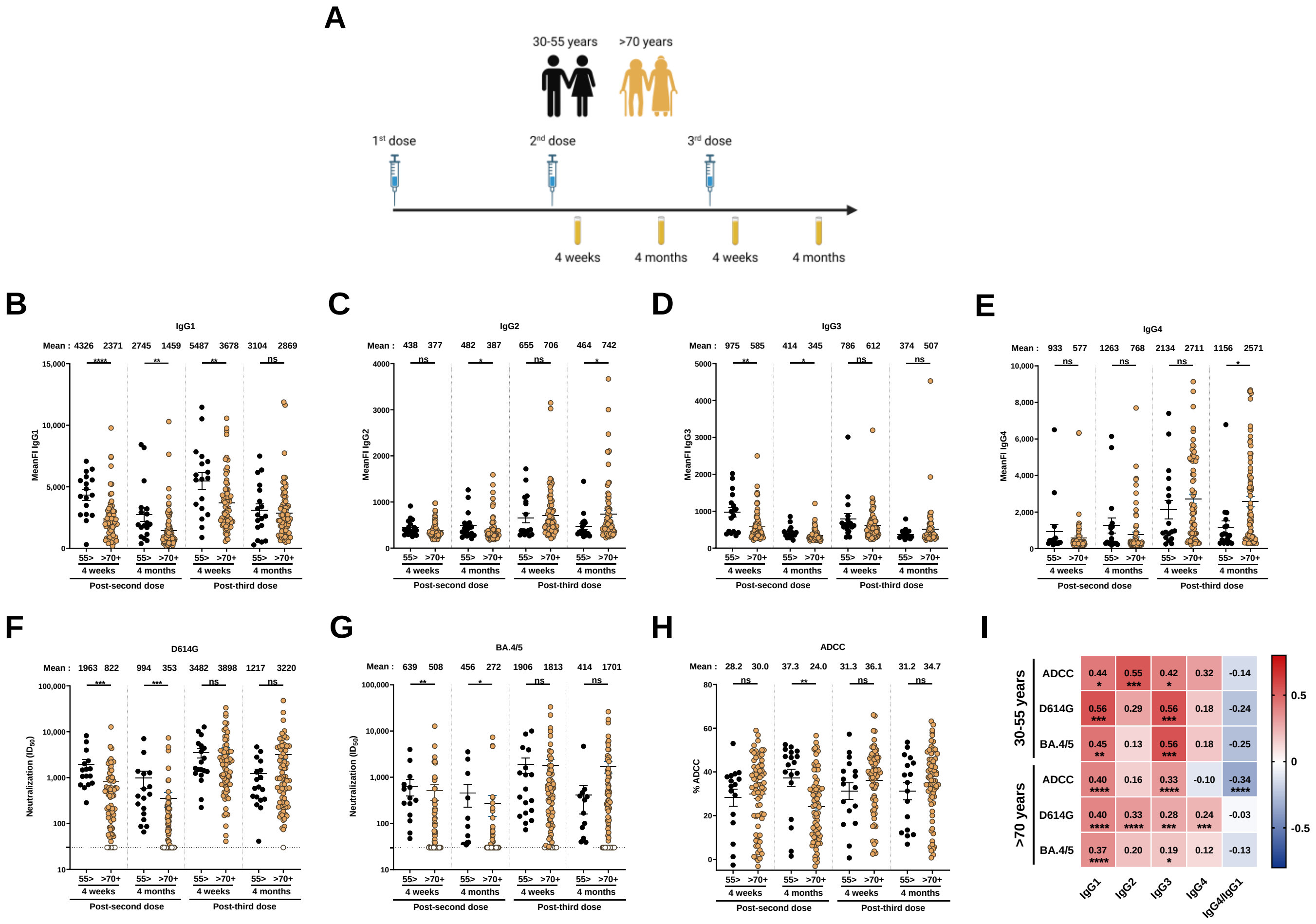

Figure S1. Humoral responses elicited after the second and third doses of mRNA vaccine in people older than 70 years compared to younger donors. (**A**) SARS-CoV-2 vaccine cohort design. Plasma samples were collected in donors aged between 30 and 55 years old and in donors older than 70 years old 4 weeks and 4 months after their second and third doses of mRNA vaccine. (**B-E**) CEM.NKr cells stably expressing the D614G Spike were stained with plasma samples and a secondary antibody allowing the specific detection of (**B**) IgG1, (**C**) IgG2, (**D**) IgG3 or (**E**) IgG4 subclasses, and analyzed by flow cytometry. The values represent the MeanFI. (**F-G**) Neutralization activities was measured by incubating viruses pseudotyped with the indicated Spike (from (**F**) D614G or (**G**) BA.4/5 variants) with plasma samples for 1h at 37°C before infecting 293T-ACE2 cells. Neutralization half maximal inhibitory serum dilution (ID_{50}) values were determined using a normalized non-linear regression. (**H**) CEM.NKr parental cells mixed with CEM.NKr cells stably expressing the D614G Spike at a 1:1 ratio (used as target cells) were incubated with plasma samples and KHYG-1 (used as effector cells) to measure ADCC activity in a FACS-based assay. (**I**) Spearman correlations between the level of the different IgG subclasses elicited after the third dose of mRNA vaccine (4 weeks and 4 months) and the functional activities of the antibodies. Plasma samples collected in donors aged between 30 and 55 years old and in donors older than 70 years are represented by black and yellow points, respectively. Undetectable measures are represented as white symbols, and limits of detection are plotted. Error bars indicate means ± SEM (∗p < 0.05; ∗∗p < 0.01; ∗∗∗p < 0.001; ∗∗∗∗p < 0.0001; ns, non-significant).

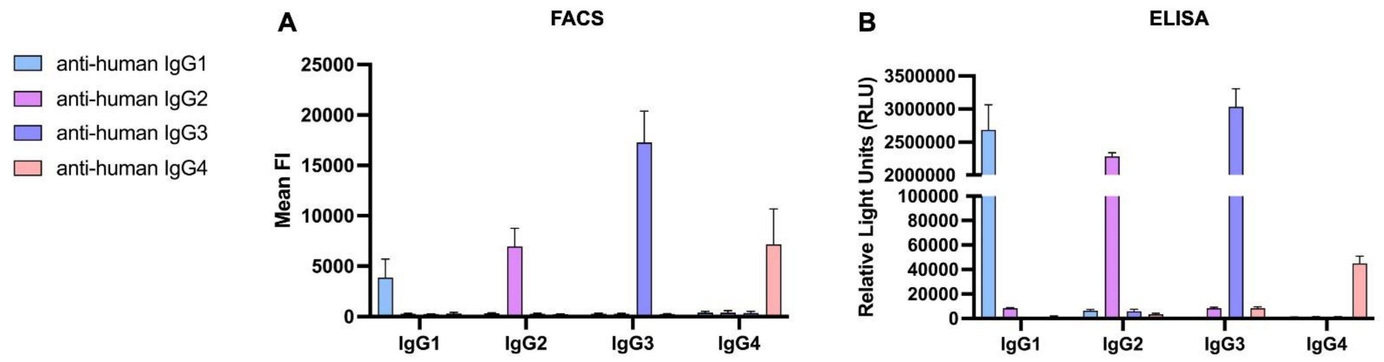

Figure S2. Binding specificity of secondary anti-IgG1, anti-IgG2, anti-IgG3 and anti-IgG4 antibodies tested by flow cytometry and ELISA. (**A**) CEM.NKr cells stably expressing the Spike glycoprotein were incubated with a primary IgG1, IgG2, IgG3 or IgG4 antibody targeting the Spike RBD. Then, unconjugated anti-IgG1, IgG2, IgG3 and IgG4 were added and incubated, followed by the staining with a tertiary anti-IgG conjugated to AlexaFluor647 and analyzed by flow cytometry (n=8). (**B**) ELISA plates were coated with the Spike and incubated with a primary human IgG1, IgG2, IgG3 or IgG4 antibody targeting the RBD. Unconjugated anti-IgG1, IgG2, IgG3 or IgG4 secondary antibody was added to the plates, followed by incubation wtih a HRP-conjugated tertiary anti-IgG antibody (n=2).

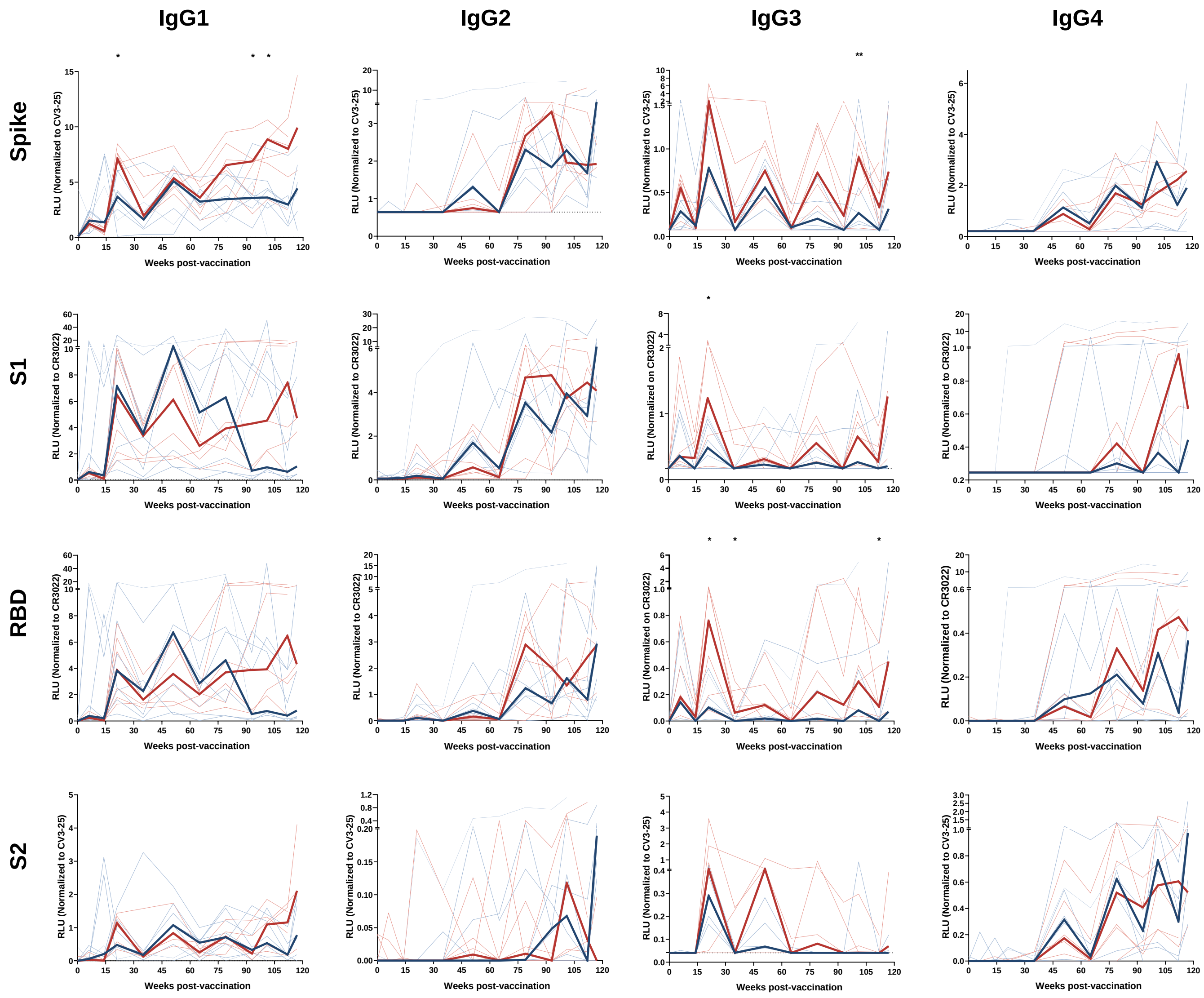

Figure S3. Levels of the different IgG subclasses elicited against different domains of the Spike in a cohort of Health Care Workers. ELISA plates were coated with the Spike, S1, RBD, or S2 and incubated with plasma from naïve or infected HCW. The different subclasses levels against each domain were measured and data were normalized to CR3022 antibody for S1 and RBD, or CV3-25 antibody for S2 and Spike. The threshold was calculated using nine pre-pandemic plasmas. Every line represents a donor. Bold lines represent the median of RLU normalized for both groups of donors. Plasma samples collected in HCW with or without BTI are represented by red and blue lines, respectively. (∗p < 0.05; ∗∗p < 0.01).

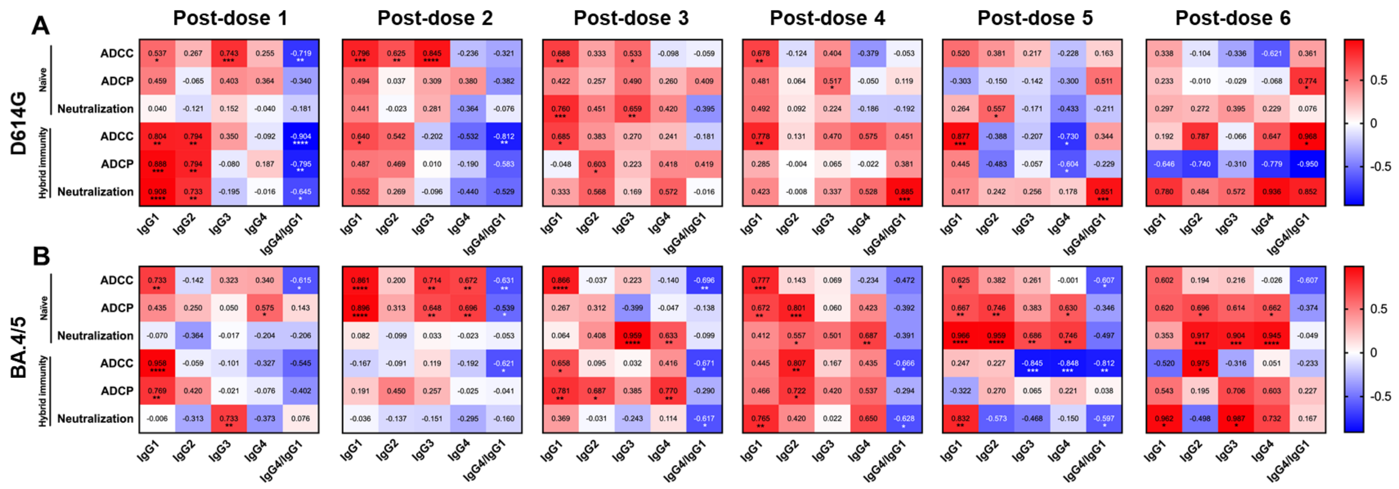

Figure S4. Spearman correlations between the levels of the different IgG subclasses elicited after every dose of mRNA vaccine and the functional activities of the antibodies, related to Figure 1. Plasma samples were collected in 14 HCW with or without a BTI 4 weeks and 4 months after every dose of mRNA vaccine. Spearman correlations between IgG subclasses and humoral responses (neutralization, ADCC and ADCP) against (**A**) D614G and (**B**) BA.4/5 were calculated using GraphPad after every dose of mRNA vaccine (4 weeks and 4 months). Values shown are Spearman r. Significance is represented by the p-value ($*p < 0.05$; $**p < 0.01$; $***p < 0.001$; $****p < 0.0001$).

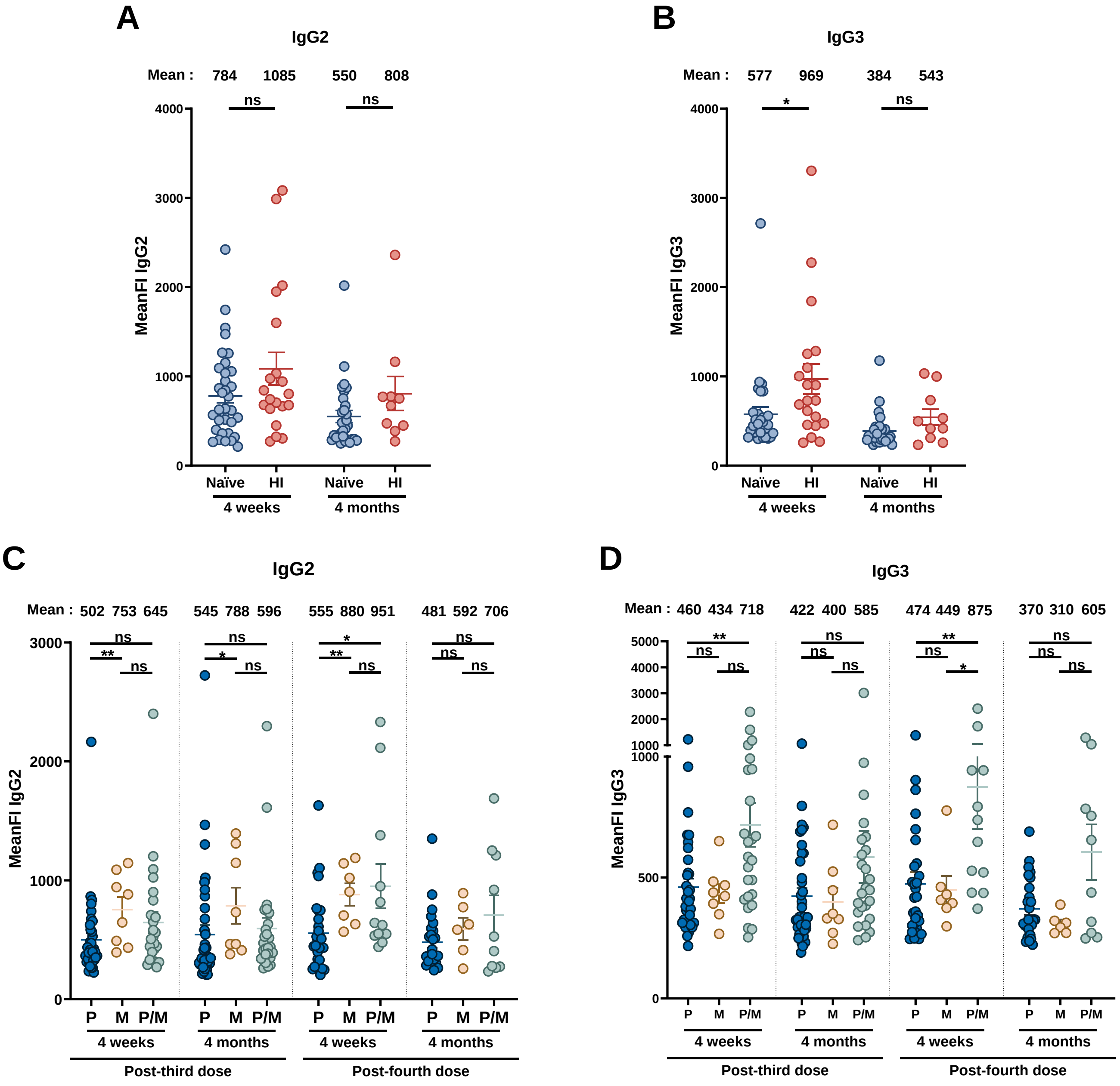

Figure S5. Levels of IgG2 and IgG3 elicited after mRNA vaccine, related to Figures 2 and 3. (**A-D**) CEM.NKr cells stably expressing the D614G spike were stained with plasma samples and a secondary antibody allowing the specific detection of (**A, C**) IgG2 or (**B, D**) IgG3 subclasses, and analyzed by flow cytometry. The values represent the MeanFI. The levels of IgG2 and IgG3 were measured in (**A-B**) donors older than 70 years old with (red points) or without (light blue points) BTI 4 weeks and 4 months after their fourth dose of mRNA vaccine (HI: Hybrid immunity), and in (**C-D**) donors older than 70 years old vaccinated with 4 doses of Pfizer (P; dark blue points), 4 doses of Moderna (M; beige points) or 2 doses of Pfizer and 2 doses of Moderna (P/M; green points) 4 weeks and 4 months after their third and fourth doses of mRNA vaccine. Error bars indicate means ± SEM (*$p < 0.05$; **$p < 0.01$; ns, non-significant).
